# Genetic Characterization of Nipah Virus, Bangladesh, 2004

**DOI:** 10.3201/eid1110.050513

**Published:** 2005-10

**Authors:** Brian H. Harcourt, Luis Lowe, Azaibi Tamin, Xin Liu, Bettina Bankamp, Nadine Bowden, Pierre E. Rollin, James A. Comer, Thomas G. Ksiazek, Mohammed Jahangir Hossain, Emily S. Gurley, Robert F. Breiman, William J. Bellini, Paul A. Rota

**Affiliations:** * Centers for Disease Control and Prevention, Atlanta, Georgia, USA; † ICDDR,B: Centre for Health and Population Research, Dhaka, Bangladesh

**Keywords:** Nipah virus, encephalitis, henipavirus, dispatch

## Abstract

Until 2004, identification of Nipah virus (NV)-like outbreaks in Bangladesh was based on serology. We describe the genetic characterization of a new strain of NV isolated during outbreaks in Bangladesh (NV-B) in 2004, which confirms that NV was the etiologic agent responsible for these outbreaks.

Nipah virus (NV) and Hendra virus (HV), the only members of the genus *Henipavirus* within the family *Paramyxoviridae* are different from most other paramyxoviruses because they have a broad host range in vivo and in vitro. Although HV and NV have genetic characteristics and replication strategies similar to those of other paramyxoviruses, the henipaviruses have several unique genetic features (*1**,**2**–**4*).

The first outbreak of NV occurred between late 1998 and early 1999 in peninsular Malaysia and Singapore and was associated with respiratory disease in swine and acute and febrile encephalitis in humans. Direct contact with sick pigs was the primary source of human infection (*1*). Of 265 human cases, 108 were fatal. Although NV is excreted in respiratory secretions and urine of patients (*1**,**5*), a survey of healthcare workers in Malaysia showed no evidence of human-to-human transmission (*6*). The reservoir of NV is presumed to be fruit bats, primarily of the genus *Pteropus* (*7**,**8*), and humans are infected through intermediate hosts such as pigs.

Recently, NV has been established as the cause of fatal, febrile encephalitis in human patients in Bangladesh during the winters of 2001, 2003, and 2004 (*9**–**12*). An NV-like virus was identified as the cause of the outbreaks in 2001 and 2003 on the basis of serologic testing (*12*). Two outbreaks consisting of 48 cases of NV were detected in 2004 in 2 adjacent districts (30 km apart) of central Bangladesh (Rajbari and Faridpur) with a case-fatality rate of nearly 75%. Because of heightened surveillance, other small clusters and isolated cases (n = 19) were identified during the same period in 7 other districts in central and northwest Bangladesh. Although antibodies to NV were detected in fruit bats from the affected areas in 2004, an intermediate animal host was not identified, which suggests that the virus was transmitted from bats to humans. Human-to-human transmission of NV was also documented during the Faridpur outbreak (*10**,**11*). We describe the genetic characteristics of 4 NV isolates from the outbreak in Bangladesh in 2004.

## The Study

Virus isolation was performed in the BSL-4 laboratory at the Centers for Disease Control and Prevention in Atlanta. Vero E6 cells were inoculated and observed for characteristic cytopathic effect, syncytium formation (*1**,**5*). NV was isolated from 2 oropharyngeal swabs (SPB200401066, SPB200406506), 1 cerebrospinal fluid (SPB200401617), and 1 urine specimen (SPB200405758) from human patients, and isolation was confirmed by reverse transcription polymerase chain reaction (RT-PCR). Two isolates were from Rajbari, and 1 was from Faridpur; the fourth isolate, from the Rajshahi district (100 km from Rajbari), was not linked to the other 2 outbreaks. The complete genomic sequence of the first viral isolate (SPB200401066) from Rajbari was derived and submitted to GenBank (accession no. AY988601) as NV-Bangladesh (NV-B). The sequences of the open reading frame (ORF) coding for nucleoprotein (N) were obtained for the other 3 isolates. The methods used for RT-PCR, sequencing, cDNA cloning, rapid amplification of cDNA ends (RACE), and sequence analysis were previously described (*2**,**3*).

The genome of NV-B is 18,252 nt in length, 6 nt longer than NV-Malaysia (NV-M), the prototype strain of NV (SPB199901924). The additional 6 nt map to the 5´ nontranslated region of the fusion protein (F) gene. The length of the NV-B genome is evenly divisible by 6, suggesting that NV-B follows the "rule of six" (*3*). The gene order and sizes of all the ORFs except V are conserved between NV-B and NV-M (Table, Figure, A). The overall nucleotide homology between the genomes of NV-B and NV-M is 91.8%, but the changes are not uniformly distributed throughout the genome. Nucleotide homologies are higher in the protein coding regions than in the noncoding regions, although the sizes of the nontranslated regions remain highly conserved (Table). The predicted amino acid homologies between the proteins expressed by NV-M and NV-B are all >92% (Table).

**Table:** Comparison of gene sequences NV-Malaysia and NV-Bangladesh*

Gene	Virus	Open reading frame			5´ nontranslated		3´ nontranslated	
		Length†	% amino acid identity‡	% nucleotide homology‡	Length§	% nucleotide homology‡	Length§	% nucleotide homology‡
N	Malay	532	98.3	94.3	57	100	586	90.8
	Bang	532			57		586	
P	Malay	709	92.0	92.0	105	91.4	469	88.1
	Bang	709			105		469	
V	Malay	52	92.5	95.7				
	Bang	55						
W	Malay	47	100	98.5				
	Bang	47						
C	Malay	166	95.2	97.6				
	Bang	166						
M	Malay	352	98.9	93.4	100	86.0	200	83.5
	Bang	352			100		200	
F	Malay	546	98.4	93.4	284	83.1	412	79.4
	Bang	546			290		412	
G	Malay	602	95.5	93.0	233	75.5	504	80.8
	Bang	602			233		504	
L	Malay	2,244	98.2	93.4	153	82.4	67	80.6
	Bang	2,244			153		67	

*NV, Nipah virus. †Length in amino acids. ‡Percentage amino acid identity or nucleotide homology after sequences were aligned by using GAP from GCG. §Length in nucleotides. NV-Malaysia gene lengths and 5´ and 3´ nontranslated sequences and gene lengths were obtained from GenBank accession no. AF212302. The percentage amino acid identity does not take into consideration conserved amino acid changes.

**Figure:**
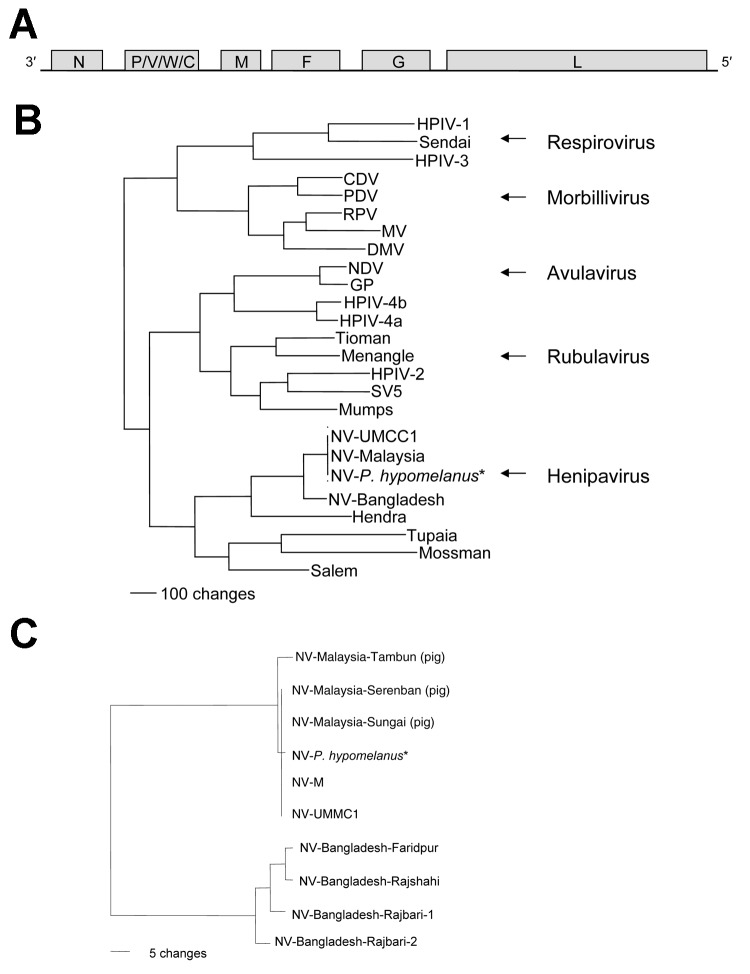
A) Schematic representation of the genome of Nipah virus (NV). Negative-sense genomic RNA is shown in 3´ to 5´ orientation. Open reading frames (ORFs) are indicated by shaded boxes: N, nucleoprotein; P, phosphoprotein; M, matrix protein; F, fusion protein; G, attachment protein; L, polymerase protein. B) Phylogenetic analysis of the N ORFs from members of the subfamily Paramyxovirinae. Arrows identify the 5 genera. A phenogram of the N ORFs of members of this subfamily was created by using maximum parsimony analysis with PAUP 4.02 (Sinauer Associates, Sunderland, MA, USA). Abbreviations and accession numbers: HPIV-1, human parainfluenza virus, D01070; Sendai, X00087; HPIV-3, D10025; CDV, canine distemper virus, AF014953; PDV, phocine distemper virus, X75717; RPV, Rinderpest virus, X68311; MV, K01711; DMV, dolphin Morbillivirus, X75961; NDV, Newcastle disease virus, AF064091; GP, goose paramyxovirus, AF473851; HPIV-4b, M32983; HPIV-4a, M32982; Tioman, AF298895; Menangle, AF326114; HPIV-2, M55320; Simian virus 5 (SV5), M81442; Mumps, D86172; NV-UMCC1, AY029767; NV-Malaysia, AF212302; NV-P. hypomelanus,* AF376747; NV-Bangladesh; Hendra virus, AF017149; Tupaia paramyxovirus, AF079780; Mossman virus, AY286409; and Salem virus, AF237881. C) The phylogenetic relationship between the N gene sequences of the 4 human NV isolates from the Bangladesh outbreak in 2004 and the N gene sequences from pig and human NV isolates from Malaysia. Accession numbers for the pig isolates of NV (*13*) are AJ627196, AJ564622, and AJ564621. *NV-P. hypomelanus is sequence from a virus isolated from Pteropus hypomelanus, the Island Flying Fox (*8*).

Overall, the predicted amino acid homologies of the surface glycoproteins, F and G, of NV-B and NV-M are high (Table). In the F protein, the predicted cleavage site, F1 amino-terminal domain, transmembrane domain, and predicted N-glycosylation sites are identical in NV-B and NV-M (*2*). Four of the 9 predicted amino acid changes occur in the first 11 amino acids (aa) of the precursor of the F protein, F0, which fall within the predicted signal peptide and would be cleaved from the mature protein. Within the G proteins, the predicted transmembrane domains, and the positions of all 17 cysteine residues are conserved between NV-B and NV-M. Of 8 predicted N-linked glycosylation sites in the G protein of NV-M, 6 are conserved in NV-B and in HV (*2*).

The coding strategy of the P gene is identical in NV-B and NV-M. In these viruses, the P gene contains the C, V, and W ORFs in addition to the P ORF. Like most other paramyxoviruses, the henipaviruses have a conserved AG-rich region that acts as an editing site to facilitate the addition of nontemplated G residues into the transcripts of the P gene. The edited transcripts encode 2 proteins, V and W, which are co-amino-terminal with P but have unique carboxy termini (*2*). The addition of 1 G residue generates the mRNA for the V protein, and the addition of 2 G residues produces the mRNA for the W protein. Sequence analysis of multiple cDNA clones containing the editing site of NV-B identified edited transcripts that encoded both the V and W proteins (data not shown). The conserved 20-nt region encompassing the editing site is identical in NV-M, HV, and measles virus (MV) (*2*); however, the editing of site of NV-M (UGGGUAAUUUUUCCCGUGUC) differs from NV-B (GGGAUAAUUUUUCCCGUGUC) at 2 nt positions (underlined). The functional significance of these 2 substitutions is under investigation. All of the cysteine residues are conserved in the V proteins of NV-B and NV-M; however, the unique portion of the V protein of NV-B is predicted to be 55 aa, 3 aa longer than the V protein of NV-M. The predicted W protein of NV-B is identical in size and sequence to the W protein of NV-M. Recently, aa 100–160 and 230–237 of the V protein of NV-M have been identified as necessary for inhibition of interferon signaling (*14*). These regions are highly conserved in the V protein of NV-B, which has 4 predicted amino acids substitutions (1 conservative) between positions 100–160 and no predicted substitutions between positions 230–237. In addition, the V protein of NV-B has 3 predicted amino acid substitutions in the CRM1-dependent nuclear export signal that was identified between aa 174–193 in the V protein of NV-M (*14*). The biologic effects of these amino acid substitutions are under investigation.

The L proteins of NV-B and NV-M had a high level of predicted amino acid conservation (Table). The 6 highly conserved domains of viral polymerases, originally described by Poch et al. (*15*) and delineated for NV-M by Harcourt et al. (*3*), remain largely unchanged between NV-B and NV-M. Domains 1, 2, and 5 have 1 conservative amino acid change each and domain 3, which is considered the most conserved domain within the L proteins of paramyxoviruses, has 2 aa changes. The 4 motifs identified in domain 3, including the QGDNE motif, which is assumed to be the active site of the polymerase, are identical between the NV-B and NV-M, as is the predicted nucleotide-binding motif in domain 6.

The cis-acting control sequences are highly conserved in the genomes of NV-B and NV-M. As in NV-M, the intergenic sequences in NV-B are GAA, with the exception of the sequence between the G and L genes, UAA, which is unique among the henipaviruses. However, the intergenic sequence between the G and L genes is GAA in the second isolate from Bangladesh. The transcriptional start and stop signals of each gene of NV-B are highly conserved in relation to the other henipaviruses. The 3´ leader sequence of NV-B is identical in length to those of all other paramyxoviruses and has nucleotide changes at positions 14 and 47 compared to NV-M. The 5´ trailer of NV-B is identical in length and sequence to NV-M.

Phylogenetic analysis was used to compare the sequence of the N ORF of NV-B to the sequences of the N ORFs from other members of the subfamily *Paramyxovirinae*. The results confirmed the results of the sequence comparisons, which show that NV-B is most closely related to the henipavirus NV-M, and support the conclusion that NV-B should be regarded as new strain of NV (Figure, B). Phylogenetic analyses conducted with the sequences of the other genes produced similar results (data not shown). The sequences of the N ORFs of 4 NV isolates from Bangladesh share 99.1% nt homology (Figure, C) but exhibited more interstrain nucleotide heterogeneity than the sequences of the human isolates in Malaysia, which were nearly identical (*1**,**8**,**13*). These varying amounts of genetic variability may reflect differences in the mode of transmission of NV in the 2 countries. In Malaysia, molecular evidence suggests that at least 2 introductions of NV into pigs occurred (Figure, C). However, the nearly identical sequences of human and pig isolates from the later phase of the outbreak suggest that only 1 of the variants spread rapidly in pigs and was associated with most human cases (*13*). In contrast, the sequence heterogeneity observed in Bangladesh may be the result of multiple introductions of NV into humans from different colonies of fruit bats.

## Conclusions

This first look at strain variation in NV indicates that viruses circulating in different areas have unique genetic signatures and suggests that these strains may have coevolved within the local natural reservoirs. Until 2004, identification of NV outbreaks in Bangladesh had been based only on serologic testing. The isolation and genetic characterization of NV-B confirm that NV was the etiologic agent responsible for these outbreaks.

Note: After this article was accepted for publication, Nipah virus was isolated from *Pteropus lylei* in Cambodia (*16*). Phylogenetic analysis of the N gene sequences demonstrated that this virus is more closely related to Nipah-Malaysia than to Nipah-Bangladesh and represented another lineage of Nipah virus.
